# Attribution of Intentional Causation Influences the Perception of Observed Movements: Behavioral Evidence and Neural Correlates

**DOI:** 10.3389/fpsyg.2013.00023

**Published:** 2013-01-29

**Authors:** James W. Moore, Christoph Teufel, Naresh Subramaniam, Greg Davis, Paul C. Fletcher

**Affiliations:** ^1^Department of Psychiatry, Brain Mapping Unit, University of CambridgeCambridge, UK; ^2^Department of Psychology, Goldsmiths, University of LondonLondon, UK; ^3^Department of Psychology, University of CambridgeCambridge, UK; ^4^Behavioural and Clinical Neurosciences Institute, University of CambridgeCambridge, UK; ^5^Cambridge and Peterborough NHS Foundation TrustCambridge, UK

**Keywords:** agency, causality, intention, mental-state attribution, intentional binding, consciousness, fMRI, social perception

## Abstract

Recent research on human agency suggests that intentional causation is associated with a subjective compression in the temporal interval between actions and their effects. That is, intentional movements and their causal effects are perceived as closer together in time than equivalent unintentional movements and their causal effects. This so-called intentional binding effect is consistently found for one’s own self-generated actions. It has also been suggested that intentional binding occurs when observing intentional movements of others. However, this evidence is undermined by limitations of the paradigm used. In the current study we aimed to overcome these limitations using a more rigorous design in combination with functional Magnetic Resonance Imaging (fMRI) to explore the neural underpinnings of intentional binding of observed movements. In particular, we aimed to identify brain areas sensitive to the interaction between intentionality and causality attributed to the observed action. Our behavioral results confirmed the occurrence of intentional binding for observed movements using this more rigorous paradigm. Our fMRI results highlighted a collection of brain regions whose activity was sensitive to the interaction between intentionality and causation. Intriguingly, these brain regions have previously been implicated in the sense of agency over one’s own movements. We discuss the implications of these results for intentional binding specifically, and the sense of agency more generally.

## Introduction

Hume famously argued that causality cannot be perceived directly but must be inferred based on certain cues such as the temporal contiguity of events (Hume, 1739/1888). According to this view, time provides the bottom-up perceptual input to the formation of higher-level causal representations. Intriguingly, more recent research on human agency implies that the reverse relationship also exists, i.e., a belief about a causal relationship between two events alters the temporal experience of those events by top-down modulation. In particular, it has been demonstrated that, when an agent is (or believes she is) the cause of an event, this causal representation can shape the way in which the timing of actions and outcomes are perceived: intentional actions, such as an active key press, and their effects, such as a tone, are perceived as closer together in time than equivalent unintentional (passive) movements and their effects (Haggard et al., 2002; Moore and Obhi, 2012). The existence of this “intentional binding” effect indicates that intentional causation is associated with the subjective binding together in time of actions and their effects. Although this intentional binding effect has been repeatedly observed in the context of voluntary action, it should also be noted here that there is ongoing debate over whether or not this effect is *specific* to voluntary action, or a property of causation more generally (Buehner, 2012; Moore and Obhi, 2012). Nevertheless, the effect reveals an intriguing reversal of the Human relationship between time and causality.

As noted above, intentional binding is consistently found for one’s own self-generated actions. However, it has also been suggested that intentional binding occurs for other people’s movements. For example, Wohlschläger et al. (2003) demonstrated that observers perceived the interval between an experimenter’s movement and its consequence as bound together in time, whereas there was no intentional binding effect for observed machine-generated movements. Assuming that observers attributed intentionality to the experimenter’s but not to the machine’s movement, these results suggest that intentional binding may be a property of intentional causation in general rather than being restricted to self-generated movements. This in turn would imply that the high-level conceptualization of an observed movement in terms of the underlying intention and causation shapes the lower-level perceptual processing of this stimulus. Whereas less is known about the role of attributed causation in perception, the notion that the attribution of mental states to a socially relevant stimulus might lead to top-down modulation of perceptual information-processing is consistent with a small but growing body of findings in the social perception literature (e.g., Teufel et al., 2009).

As indicated above, previous studies focusing on first- and third-person intentional binding suggest that, at the perceptual level, there is a distinction between intentional and unintentional causation (but, see Buehner, 2012). The purpose of our study was to add to this literature by exploring brain areas sensitive to the interaction between intentionality and causality when observing other people’s movements. In order to be able to address this question, we extended the paradigm used by Wohlschläger et al. (2003) to overcome two limitations. Firstly, the perceptual input used in this study was not matched across the human and machine conditions: in the former, participants saw the movements of the experimenter’s gloved hand, while in the latter, they saw a disembodied rubber hand being pulled down by a mechanical device. Such perceptual differences preclude clear distinctions between top-down and bottom-up influences on perception because differences in bottom-up input are confounded with potential top-down effects. In other words, it is impossible to tell whether perceptual differences between conditions rather than the observer’s beliefs regarding the intentionality or causality of the movements might be responsible for the differences in perceived duration between an observed movement and its outcome. A second caveat pertaining to the Wohlschläger et al. study is that the key conditions were distinguished not just according to intention but also the presence of an agent: a human hand, unlike a rubber hand operated by a machine, belongs to an agent. In this way, “intentionality” of the stimulus was not systematically and exclusively manipulated.

Our paradigm ensured that (i) sensory stimulation was identical in the different attribution conditions, (ii) with respect to mental-state attribution only intentionality was manipulated, and (iii) we could, on a neuronal level, tease apart the individual and combined effects of attributed intentionality and causation. Participants viewed simple key press movements that caused a tone outcome. Due to an elaborate deception procedure, observers believed that these movements were either intentional or forced upon the finger of the other person, i.e., unintentional. Crucially, the stimuli and thus the bottom-up inputs were perceptually identical across conditions. Using this paradigm, we assessed binding behaviorally with the interval estimation procedure (see Moore and Obhi, 2012, for review). In order to be able to tease apart the individual and combined roles of attributed intentionality and causation using functional Magnetic Resonance Imaging (fMRI), we included two additional non-causal conditions. That is, participants not only viewed (apparent) intentional and (apparent) unintentional causal movements (i.e., key presses that caused tones), they also viewed (apparent) intentional and (apparent) unintentional non-causal movements (i.e., key presses that did *not* cause tones).

Behaviorally, we predicted that, if intentional binding for observed movement does reflect the top-down role of attributed intentionality, binding should be present in intentional but not unintentional causal movements even when perceptual input was identical (as it was in our paradigm). In order to explore the neuronal correlates of the combined effect of attributed intentionality and causation on perception of the finger movements, we chose our ROIs based on two principles. First, in order to assess the extent to which first- and third-person intentional binding are underpinned by at least partly overlapping processes, we selected our ROIs based on previous fMRI investigations of first-person intentional binding (for review, see Sperduti et al., 2011). In particular we focused on the insula, supplementary motor areas, dorsolateral prefrontal cortex, angular gyrus, and superior parietal cortex. In addition, we added brain areas that have been implicated in social perception and social cognition such as medial prefrontal cortex (mPFC), temporo-parietal junction (TPJ), and superior temporal sulcus (STS). This collection of regions has been speculated to be a key network underpinning top-down effects on the perception of socially relevant information (Teufel et al., 2010).

## Materials and Methods

### Participants

Nineteen participants took part in the study (mean age: 22 years; 16 females). Three participants were excluded from the analysis. One participant failed to follow task instructions, one participant did not believe the deception (revealed during the de-brief), and one participant had uncorrected visual impairment (self-reported by participant).

The experiment was approved by the National Research Ethics Service (NRES). All participants gave informed consent prior to the experiment.

### Behavioral task

#### Design

We used a factorial design to systematically explore the effects of intentionality (“intentional,” “unintentional”) and causality (“causal,” “non-causal”) on movement perception and neural activity.

#### Pre-scanning session

**Figure 1:**
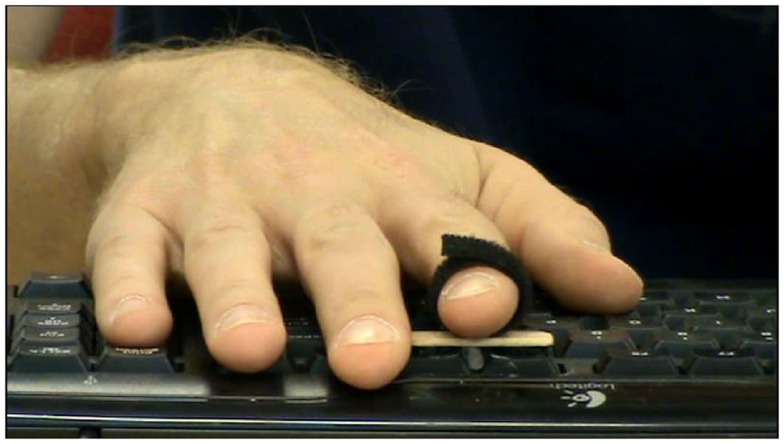
**A video still showing an example stimulus used in the experiment**. The same set of videos was used in all four conditions (intentional causal, intentional non-causal, unintentional causal, unintentional non-causal). In the causal conditions the key press made in the video caused a tone effect after a delay of 100, 400, or 700 ms. The only difference in the two causal conditions was the description of the movement and, consequently, the belief of the observer regarding its intentionality. In these causal conditions participants estimated the duration of this interval in milliseconds. In the non-causal conditions the key press did not cause a tone effect. Again, the only difference in the non-causal conditions was the description of the movement (intentional or unintentional). In these non-causal conditions the participants had to press a response key as soon as possible after they detected an asterisk appearing at a random location on the video over the model’s hand (on 20% of trials).

Participants attended a pre-scanning session in which they were shown the experimental set-up and given practice with the paradigm. The paradigm depended upon participants believing that they were watching a live webcam video-link of another person, similar to a Skype conference, when in fact they were watching pre-recorded videos (see Teufel et al., 2010, for rationale). We first showed them a phoney video-link set-up in which there was a webcam in one of two adjacent rooms. A confederate was also sat in this room. Participants were told that they would see this person – via the “live” webcam video-link – performing simple manual key press movements on a keyboard (see Figure 1). Participants also briefly interacted with the apparatus that the confederate would supposedly be using in the experiment. This apparatus consisted of a keyboard on which a response key could be actively pressed down or a harness attached to the button that could cause the finger passively to move down. Participants made one active key press and were also subjected to one passive key press.

Following this introduction, participants were taken to the adjacent room where they completed practice trials in four different conditions. In two “causal” conditions participants watched videos of the confederate’s finger moving down on the response key. This movement caused a beep after one of three delays: 100, 400, 700 ms (following Moore et al., 2009). Participants were told that the movement-beep interval randomly varied between 50 and 950 ms. We employed an interval estimation paradigm to assess the observers’ perception of the duration between the observed movement and the tone. To make their judgment they were initially presented with the default number 500 ms and were told to press a right key to increase this number (in 50 ms increments) or a left key to decrease this number (in 50 ms increments). They continued to increase or decrease the number on the screen representing their interval estimate until they indicated by a button press their final response. These two “causal” conditions differed in terms of intention attribution: in one condition participants were told that the confederate intentionally pressed the button, in the other condition they were told that their finger was passively moved by a motor. Crucially, the videos were identical in both intentional and unintentional conditions. The only difference was the description of the movement, and consequently the belief of the observers regarding the intentionality of the observed button press. Each video lasted 4 s and included a variable delay before movement onset.

In two “non-causal” conditions participants watched videos of the confederate’s finger moving but this time the movement did *not* cause a beep outcome. These non-causal conditions were included to allow us to investigate areas of brain activation that were sensitive to the interaction between intentionality and causality. In order to maintain participants’ attention to the screen in these conditions and to provide a measure of spatial attention allocation, they were given a behavioral task that required them to respond as quickly as possible to an asterisk appearing at a random location on the video of the confederate’s hand on 20% of trials. The only information participants were told about the asterisks was that their appearance was random. These two “non-causal” conditions also differed in terms of intention attribution: in one condition participants were told that the confederate intentionally pressed the button, in the other condition they were told they unintentionally pressed the button. Again, the videos were identical in both intentional and unintentional conditions. The only difference was the description of the movement. Each video lasted 4 s and included a variable delay before movement onset.

Each of the four conditions consisted of 12 trials. In the “causal” conditions (in which the movement caused the beep), there were four trials per interval duration. Conditions were blocked by intentionality. See Figure 1 for example video stimulus used in the experiment.

#### Scanning session

When participants arrived for the scanning session they were told that the same live webcam video-link was set-up as they had seen in the pre-scanning session. However, they were not shown it this time. In the scanner they completed the same four conditions they had practiced in the pre-scanning session: intentional non-causal; intentional causal; unintentional non-causal; unintentional causal. Conditions were blocked by intentionality and there were 36 trials per condition. In the “causal” conditions (in which the movement caused the beep), there were 12 trials per interval duration. All conditions were divided into blocks of six trials separated by a period of rest during which participants fixated on a cross on the screen for 12 s.

Prior to each condition the participants were told over the intercom the nature of the movement (“intentional” or “unintentional”) and whether or not the movement was causal. They were also reminded of their task, i.e., either interval estimation or detection of an asterisk.

The interval estimates in the causal conditions allowed us to measure intentional binding. We predicted lower interval estimates in the “intentional” condition vs. the “unintentional” condition (following Wohlschläger et al., 2003). The asterisk response task ensured that participants maintained their focus of attention on the moving hand in the non-causal conditions.

### Functional magnetic resonance imaging

We used a Siemens Trio scanner, operating at 3 T, with a 225 mm field of view in the Wolfson Brain Imaging Centre, Cambridge. In total, 300 volumes were acquired using a T2*-weighted echo-planar imaging sequence with 32 slices, acquired in descending order with an oblique axial orientation, covering the whole brain. Each slice was 3 mm thick with an inter-slice gap of 0.8 mm. A repetition time of 2000 ms was used with echo time; TE = 30 ms, flip angle = 78°, and matrix size 64 × 64.

Data were analyzed using statistical parametric mapping in the SPM5 program (http://www.fil.ion.ucl.ac.uk). Images were realigned then spatially normalized to a standard template and spatially smoothed with an isotropic three dimensional Gaussian filter (8 mm full width at half-maximum). The time series in each session were high-pass filtered (with cut-off frequency 1/120 Hz) and serial autocorrelations were estimated using an AR(1) model.

Four experimental conditions (intentional non-causal, intentional causal, unintentional non-causal, and unintentional causal) were modeled using a box car function convolved with a canonical hemodynamic response. Conditions were specified as covariates in a general linear model and the beta parameter estimated at each voxel for each stimulus type, derived from the mean least-squares fit of the model to the data. The responses to each condition were compared to the fixation baseline, and each of these four contrasts was taken forward to a group analysis treating inter-subject variability as a random effect.

Anatomically defined ROIs were selected based on previous fMRI studies on sense of agency (see Sperduti et al., 2011 for review). Specifically, we included: insula, supplementary motor areas, dorsolateral prefrontal cortex, angular gyrus, and superior parietal cortex. In addition, we added the following ROIs: mPFC, TPJ, and STS. This collection of regions is thought to be a key network underpinning top-down effects on social perception (Teufel et al., 2010). ROIs were specified using PickAtlas toolbox (Maldjian et al., 2003). We report significant interactions, corrected for multiple comparisons (FDR *p* < 0.05 within the mask).

## Results

### Behavioral: Intentional binding

The intentional binding effect was measured by comparing mean interval estimates in the “intentional causal” vs. “unintentional causal” conditions (following Moore et al., 2009). A behavioral study (*N* = 19) was conducted outside the scanner, using the same procedure, to examine the effect of intention attribution on intentional binding. The results showed that the mean interval estimate in the intentional causal condition was significantly lower than in the unintentional causal condition, *t*(19) = 2.22, *p* = 0.040 (two-tailed). Based on the results of this initial behavioral result coupled with Wohlschläger et al.’s (2003) study, one-tailed *t*-tests were used for the analysis of intentional binding data collecting inside the scanner. As predicted, the mean interval estimate in the intentional causal condition (542 ms) was significantly lower than in the unintentional causal condition (560 ms), *t*(15) = 1.94, *p* = 0.036 (one-tailed; see also Appendix). Although this effect is weaker than that found in the prior behavioral study, it nevertheless shows that intentional binding *does* hold for observed movements. This is consistent with Wohlschläger et al.’s (2003) results.

### Behavioral: Reaction times and error rates

We compared reaction times (RTs) to the asterisk in the two non-causal conditions as differences in RTs may indicate more general differences in the allocation of attention in the different intention attribution conditions. One participant failed to respond at all to the asterisk in the intentional non-causal and was therefore excluded from this analysis. Although there was a numerical decrease in reaction time in the intentional non-causal condition (779 ms) vs. the unintentional non-causal condition (796 ms), this difference was not statistically significant, *t*(14) = 1.73, *p* = 0.105 (two-tailed). This suggests that differences in the allocation of attention cannot explain our key result.

In order to test further the possible confounding effect of attention, we examined the relationship between RTs and intentional binding. This allowed us to determine whether or not differences in intentional binding were related to (general) differences in attention. We ran a correlation analysis on the difference in mean interval estimates (intentional causal condition vs. unintentional causal condition) and the difference in mean RTs (intentional non-causal condition vs. unintentional non-causal condition). We found no significant correlation, *r*(15) = −0.07, *p* = 0.80 (two-tailed). This suggests that putative general differences in attention (as measured by RTs) are unrelated to the intentional binding effect.

Errors of commission (pressing the response button in the absence of the asterisk) and omission (failing to press the response button in the presence of the asterisk) were also calculated. Excluding the participant who failed to respond at all to the asterisk (see above), there were no errors of commission and only two errors of omission across the entire sample.

Taken together these results suggest that overall task performance was high and that differences in attention and performance are unlikely to explain our results.

### Functional magnetic resonance imaging

#### Interaction between “intentionality” and “causality”

**Figure 2:**
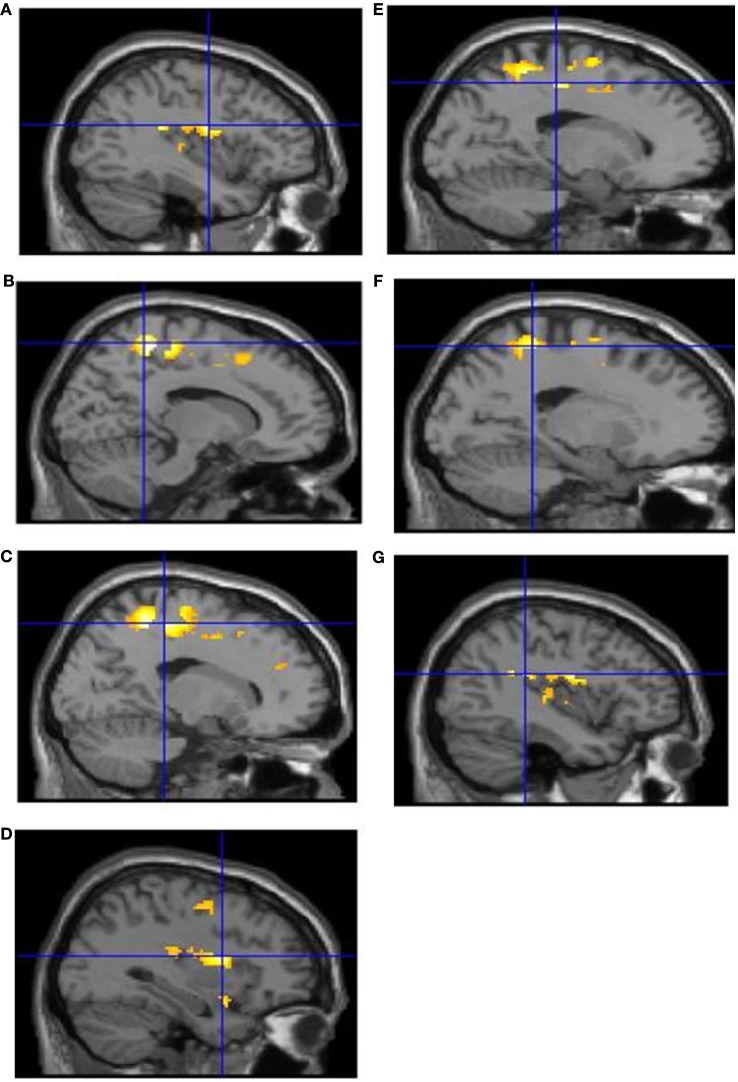
**Activations reflecting the interaction between factors of “intentionality” and “causality” from the ROI analysis in: left mid-insula (A); right superior parietal cortex (B); right motor cortex (C); right anterior insula (D); left motor cortex (E); left superior parietal cortex (F); left posterior insula (G)**.

**Figure 3:**
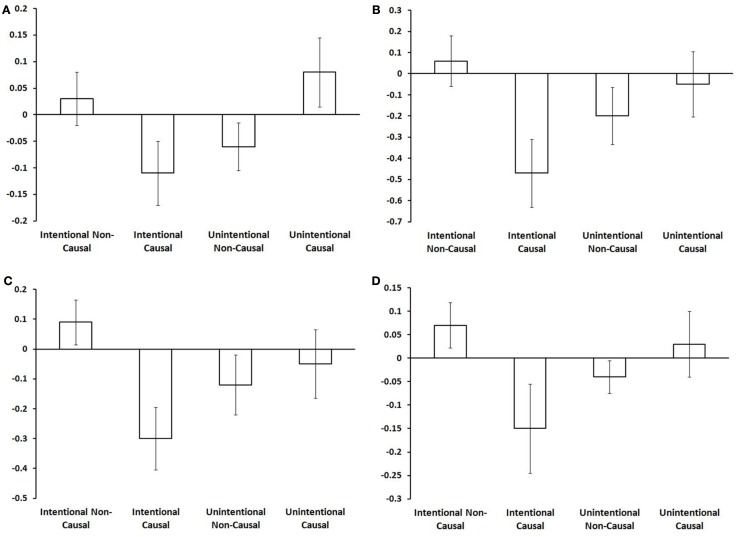

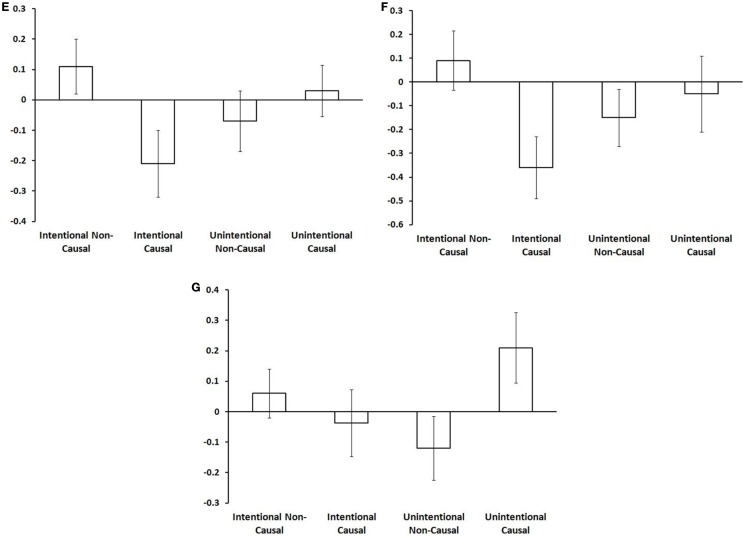
**Beta values in each area for each condition (from left to right: intentional non-causal, intentional causal, unintentional non-causal, unintentional causal)**. Although they are intended for the purposes of guidance only, these plots do suggest differences in the nature of the interaction in these regions. The full cross-over interaction is only found in the left mid-insula **(A)**. An increase in activation for intentional non-causal vs. intentional causal is found in right superior parietal **(B)**, right motor cortex **(C)**, right anterior insula **(D)**, left motor cortex **(E)**, and left superior parietal cortex **(F)**. The only area showing an increase in activation for unintentional causal vs. unintentional non-causal is left posterior insula **(G)**.

Intentional binding reflects a distinction, at the perceptual level, between intentional and unintentional causation. Using fMRI we investigated this distinction at the neural level. Specifically, we explored activations sensitive to the interaction between intentionality and causality. ROI analyses highlighted the involvement of a collection of brain regions reflecting this interaction (see Table 1). These activations are shown in Figure 2 and the associated beta values are shown in Figure 3. Superior parietal cortices and motor cortices showed reduced activations for intentional causal vs. intentional non-causal conditions (see Figures 2B,C,E,F and 3B,C,E,F). A more complex picture is found within the insula. Like superior parietal and motor cortices, reduced activation was found in right insula for intentional causal vs. intentional non-causal conditions (see Figures 2D and 3D). On the other hand, activation in left posterior insula was increased in the unintentional causal vs. unintentional non-causal conditions (see Figures 2G and 3G). Finally, the left mid-insular showed the full cross-over interaction, that is, reduced activations for intentional causal vs. intentional non-causal conditions and increased activation in the unintentional causal vs. unintentional non-causal conditions (see Figures 2A and 3A). The specific directions of these effects are scrutinized in the Section “Discussion.”

**Table 1:** **Activations reflecting the interaction between factors of “intentionality” and “causality” from the ROI analysis**.

Area	Side	*X*	*Y*	*Z*	*Z*-score
Mid-insula	L	−38	−5	21	4.37
Anterior insula	R	34	16	14	4.06
Posterior insula	L	−40	−34	22	3.76
Superior parietal	R	14	−41	60	4.29
Superior parietal	L	−18	−38	57	3.81
Primary motor cortex	R	18	−28	53	4.10
Primary motor cortex	L	−16	−23	49	3.84

*Talairach co-ordinates are reported*.

The main effect analysis for “intentionality” revealed no significant activations in our regions of interest. The main effect of “causality” was not analyzed owing to confounding task differences between the causal and non-causal conditions.

It should be noted that, based on the neuroimaging literature in social perception, Teufel et al. (2010) speculated about the involvement of mPFC, TPJ, and STS in mediating these top-down effects on action perception. Therefore, our failure to find significant activations in this network for both the interaction and main effect analyses was surprising. The possible reasons for this are considered in the Section “Discussion.”

## Discussion

Intentional binding refers to the subjective compression of time between intentional movements and their causal consequences. This effect has been most consistently observed for self-generated movements. However, here we report evidence that intentional binding also occurs for observed movements of another person; this third-person intentional binding effect is consistent with previous findings (Wohlschläger et al., 2003). Moreover, using fMRI we explored, at the neural level, the distinction between intentional and unintentional causation that is thought to underlie intentional binding and was hypothesized to be equally important for binding of observed movements. Consistent with our predictions, we found that a collection of brain regions thought to underlie intentional binding of one’s own actions was also sensitive to the interaction between attributed intentionality and causality for observed movements. Taken together, the findings indicate (i) that third-person intentional binding is a top-down effect of higher-order areas on lower-level perceptual areas, (ii) that intentional binding relies on intentional causation but is not self-specific, and (iii) that first- and third-person intentional binding are most likely subserved by at least partly shared mechanisms. We discuss these issues in more detail in the following sections.

### Top-down effects on social perception

As mentioned in the Section “Introduction,” previous work on intentional binding for observed movements (e.g., Wohlschläger et al., 2003) is undermined by limitations with the paradigm used to study this phenomenon. Most problematic for the question regarding the source of these effects is that perceptual input differed in the two attribution conditions. This is a serious limitation because it makes it impossible to separate bottom-up from top-down effects on this task. In other words, any difference in third-person intentional binding between the two attribution conditions might be due to differences in the perceptual input rather than an effect of top-down modulation by attributed intentionality or agency.

In order to address this issue, we explored intentional binding for observed movements using a more rigorous paradigm (Teufel et al., 2010), in which sensory stimulation was identical in the different attribution conditions; the only difference between them was whether observers believed that the movement they viewed was generated intentionally or was forced upon the finger of the other person. The fact that, even with this design, we found an increase in third-person intentional binding when participants attributed an intention to the movement compared to when they believed it was unintentional indicates that this effect cannot be due to differences in bottom-up input. Therefore, it is most likely a result of a top-down modulatory influence of intention attribution on those processes that underlie the perceptual binding of observed actions and their consequences. This finding adds to a number of recent studies indicating that the higher-level conceptualization of a stimulus in terms of the underlying mental states can shape lower-level social information-processing.

In a recent proposal by Teufel et al. (2010), the authors speculated about the neural implementation of such top-down modulation of perceptual processing by the high-level conceptualization of the stimulus in terms of mental states. In particular, they proposed that the high-level component is localized in the theory of mind network, including mPFC (e.g., Fletcher et al., 1995) and TPJ (e.g., Saxe and Kanwisher, 2003), whereas perceptual processing of biological motion and other social stimuli seems to primarily take place in STS (e.g., Puce and Perrett, 2003). In neural terms, top-down modulation of perception by mental-state attribution would thus imply a feed-back loop between mPFC/TPJ and STS. A recent study provided some support for this hypothesis with respect to automatic imitation, the tendency of an observer to automatically mimic the movement of another person (Wang et al., 2011). Given that automatic imitation is closely linked to action perception – in fact, in the proposal by Teufel et al. (2010), the effects on imitation are a knock-on effect of the modulation of perception – it is surprising that we did not detect a similar influence of the ToM network on perceptual processing of another person’s movements in the current study. Even more surprising is our failure to detect increased activation of these areas when participants attributed an intention to the observed movements compared to when they believed the movements were unintentional. This lack of activation of crucial parts of the ToM network is inconsistent with many previous reports and necessitates further consideration in future studies.

One possible reason for the lack of activation in this network is that our paradigm was not sensitive enough to produce these activations. However, we think this is unlikely given the fact that (a) we found significant activations in different brain regions for the interaction analysis, and (b) there was a difference in intentional binding in the intentional and unintentional conditions. Both findings suggest that our manipulations were effective. In light of this, we would suggest that this lack of activation may be linked to important differences between previous studies on intention attribution and the current one. First, whereas in previous studies the observed action was typically not followed by any obvious outcome, in our study, both the intentional and the unintentional movements were causal determinants of a tone. While this is largely speculation, it might turn out that the brain processes movements with and without obvious outcomes differently. A second difference relates to the fact that previous studies have used perceptually different stimuli in the different conditions. Conceptually, these stimulus differences are supposed to signal to the observer differences in intentionality of the movements. In our study, the differences in intentionality were not perceptually signaled but were only present in the way in which the observer conceptualized the stimuli (due to our deception procedure). A possible explanation for a lack of activation in ToM related areas in our study might be that attributed intentions that are signaled by the bottom-up input are processed differently than attributed intentions that are purely set-up by the observer’s belief system. A more controversial interpretation of previous studies is that because the intention conveyed by a stimulus and the perceptual properties of the stimulus were confounded, it might be that increased activation in mPFC, TPJ, and STS directly reflect differences in perceptual processing rather than reflecting the attribution of intentions.

### The neurocognitive basis of intentional binding, agent causation, and lack of self-specificity

Although intentional binding is a widely used implicit measure of sense of agency, there is, nevertheless, an ongoing debate about what processes intentional binding reflects (Moore and Obhi, 2012). For example, some have suggested that intentional binding is not a specific property of agent causation, but is instead a property of any causal relationship. Indeed, a number of studies have demonstrated the importance of causality for intentional binding (Buehner and Humphreys, 2009; Moore et al., 2009; Buehner, 2012). The current findings, although not ruling out the role of causality, do at least suggest that the presence of intentionality augments binding. These results also suggest that whilst binding is likely to be augmented for intentional agent causation, this effect is not *self*-specific. That is, intentional binding is not only found for one’s own self-generated movements but instead appears to be a more general property of agent causation. This raises important questions regarding the neurocognitive processes supporting binding and whether they are the same for first-person and third-person binding.

It has been suggested that sensorimotor processes play a central role in intentional binding (Haggard et al., 2002). This is based on observations that intentional binding is most consistently found for voluntary actions (i.e., those which necessarily engage sensorimotor processes; see Moore and Obhi, 2012, for a review). This assumption is potentially undermined by our findings, which show intentional binding occurs when people are *passively* observing another person move. Here, the motor system of the observer is not overtly engaged.

One possible explanation for this finding is that sensorimotor information is not essential for the intentional binding effect. Indeed, this has been demonstrated by a number of studies showing that binding can occur in the absence of voluntary movement. For example, by modifying intentional content prior to a passive movement (Moore et al., 2009) or by *implying* self-causation prior to a passive movement (Dogge et al., 2011), one can modulate the magnitude of binding. This is consistent with a recent theoretical framework highlighting the contribution (and optimal integration) of various cues to sense of agency, of which sensorimotor information is just one (e.g., Moore and Fletcher, 2012).

Another possible explanation is that, although the sensorimotor system is not overtly engaged during action observation, it is nevertheless *covertly* activated. This could generate the binding effect for observed movements. Our fMRI data offer indirect support for this hypothesis. We selected a number of ROIs based on regions commonly involved in the sense of agency of *one’s own* overt actions. In the present study we found that a number of these regions were also involved in discriminating between intentional and unintentional causation when *observing* someone else move, including superior parietal cortices, the insula, and primary motor cortices. The involvement of these regions, in particular the primary motor cortices, suggests that sensorimotor processes engaged when performing an action also contribute to agency processing when observing an action. This hypothesis is supported by a large body of research highlighting the tight link between systems involved in action execution and action observation. For example, when observing someone else move there is an automatic tendency to imitate these movements (Brass et al., 2001). Moreover, this tendency is influenced by higher-level mental-state attributions. For example, Wang et al. (2011) found that automatic imitation was enhanced during direct eye contact, and Liepelt et al. (2008) found that it was enhanced when people were led to believe the movements they were seeing were intentional. This latter study is particularly relevant and offers a plausible explanation for our finding of increased binding when people were led to believe the action was intentional: this instruction would have increased covert sensorimotor activity during action observation.

### Patterns of activation: The role of prediction error?

There was an intriguing pattern of activation in those regions reflecting the distinction between intentional and unintentional causation (see Figure 3). The interactions revealed a relative increase in activity in both unintentional causal and intentional non-causal conditions. We can only offer a speculative account of what this may mean. One possibility is that these activations are linked to prediction error. Central to this proposal is the notion that action and goal/outcome representations are inextricably linked. According to so-called “response-outcome” (R-O) theories of intentional action (Thorndike, 1931; Dickinson and Balleine, 1993, 1994; de Wit and Dickinson, 2009), once R-O associations have been established, thoughts about actions prior to movement automatically trigger thoughts about associated outcomes. These outcomes are then evaluated with respect to goals and the appropriate response is selected. Based on the assumption of a shared network for action generation and action perception, and in line with R-O theories, we would suggest that when participants were led to believe they were watching an *intentional* action, this would first activate the shared action network, which in turn automatically activates an outcome representation. The higher activity for intentional non-causal action vs. intentional causal action may represent error-related increases in activation linked to the *absence* of an *expected* effect. Regions that appear to be particularly sensitive to this include: superior parietal cortex, the motor cortex, and the right insula. Following this same logic, the representation of *unintentional* action should *not* activate goal/outcome representations. If this were the case then when one is led to believe they are watching an unintentional action, this would fail to activate goal/outcome representations. The higher activity for unintentional causal action vs. unintentional non-causal action may also be error-related activation linked to the *presence* of an *unexpected* effect. The single region that is particularly sensitive to this is the left posterior insula.

Although speculative, this prediction error hypothesis receives support from previous studies which demonstrate the involvement of these regions in outcome prediction and/or the encoding of prediction error. For example, it is well established that the parietal lobe is involved in sensorimotor prediction (Andersen and Buneo, 2002; Blakemore and Sirigu, 2003). Furthermore, it has been shown activity within superior parietal regions is higher during *unpredictable* externally produced tactile stimulation compared with predictable self-produced tactile stimulation (Blakemore et al., 1998). The insula, another core region highlighted by our analyses, is also commonly activated when predictions are violated (Preuschoff et al., 2008; Bossaerts, 2010). Of particular relevance is the suggestion that performance monitoring – detecting mismatches between goals and outcomes – is one of the primary functions of the insula (and in particular, the anterior insula; Ullsperger et al., 2010).

## Conclusion

In summary, our findings support a number of conclusions. First, the fact that intentional binding not only holds for self-generated but also for observed movements suggests that, although it may be a property of *agent* causation, it is not self-specific. Second, we were able to establish the presence of intentional binding for observed movements in the absence of perceptual differences between intentional and unintentional condition. This represents an important methodological advance. Finally, our fMRI data reveal a collection of regions whose activity reflects the interaction between intentionality and causality, something that lies at the heart of the intentional binding effect. These regions have also been implicated in the sense of agency over one’s own movements. In light of these observations we have suggested that common mechanisms may underpin the experience of self-agency and the attribution of agency to others.

## Conflict of Interest Statement

The authors declare that the research was conducted in the absence of any commercial or financial relationships that could be construed as a potential conflict of interest.

## Appendix

**Table A1:** **Average interval estimate (ms) for each interval length in the intentional causal and unintentional causal conditions**.

	100 ms	400 ms	700 ms
Intentional causal	354 (26)	569 (21)	705 (22)
Unintentional causal	348 (26)	596 (26)	739 (22)

*SEM in parentheses*.

*A 2 (intentionality: intentional causal/unintentional causal) × 3 (interval length: 100/400/700 ms) repeated measures ANOVA conducted on these data (Table A1) shows a near-significant main effect of “intentionality,” *F*(1, 15) = 3.75, *p* = 0.072, a significant main effect of “interval length,” *F*(2, 30) = 137.73, *p* < 0.001, and no significant interaction between these factors, *F*(2, 30) = 2.01, *p* = 0.15*.
